# Practitioners’ perspectives on evaluating treatment outcomes in traditional Chinese medicine

**DOI:** 10.1186/s12906-017-1746-8

**Published:** 2017-05-18

**Authors:** Yan-Hong Zhang, Jing Lv, Wei Gao, Jun Li, Ji-Qian Fang, Li-Yun He, Bao-Yan Liu

**Affiliations:** 1 Institute of Basic Research in Clinical Medicine, China Academy of Chinese Medical Sciences, Dongzhimennei Nanxiaojie #16, Beijing, 100700 China; 2 Guang’anmen Hospital, China Academy of Chinese Medical Sciences, Beixiange #5, Beijing, 100053 China; 3 Ba Li Zhuang Community Health Service Center, Yanjingxili #11, Beijing, 100025 China; 4 China Institute of History and Literature of Chinese Medicine, China Academy of Chinese Medical Sciences, Beijing, 100700 China; 5 School of Public Health, Sun Yat-sen University, 74 Zhongshan Road II, Guangzhou, 510080 China; 6 China Academy of Chinese Medical Sciences, Beijing, 100700 China

**Keywords:** Traditional Chinese medicine, Treatment outcomes, Evaluation, Practitioners

## Abstract

**Background:**

There are no generally accepted standards for evaluation of treatment outcomes in traditional Chinese medicine (TCM). Pattern differentiation and individual treatments are recognized as the most distinguishing features of TCM. Therefore, how practitioners determine curative effects is an issue worthy of research, though little has been done in this area up to this point. This study examines perceptions of the effectiveness of TCM treatments and the means of evaluating clinical outcomes from the practitioners’ perspective.

**Methods:**

Qualitative analysis of semi-structured interviews.

**Results:**

A total of nine TCM practitioners from three university-affiliated hospitals and two scientific institutions participated in the interviews in August 2013. Participants reported evaluation of periodical treatment as an important part of the process of individual treatment based on pattern differentiation. Themes included (1) ways of evaluating treatment outcomes; (2) relationships between treatment outcomes and pathological transformation; and (3) distinguishing manifestations of the healing process from true adverse reactions. These considerations helped determine the optional treatment principles for further follow-up. An additional theme emerged related to the characteristics of diagnosis and treatment in TCM.

**Conclusions:**

Health professionals considered all of the following as important ways of evaluating TCM treatment outcomes: patients’ input and subjective experience, physicians’ intake and examination, laboratory tests and medical device measurements. Pathological conditions were determined based on all the above factors, and no single factor determined the effectiveness from the practitioners’ perspectives. If the patients felt no significant beneficial effects, then it was necessary to judge the effectiveness from adverse effect. The follow-up measures were usually based on the previous treatment, and physicians’ satisfaction with each phase of TCM treatment was a significant factor in the process of making further decisions.

## Introduction

Determining appropriate methodologies for evaluating treatment outcomes in traditional Chinese medicine (TCM) is a problem that has long perplexed researchers in this area. This is mainly due to the fact that the methodologies used in TCM cannot be easily described in terms that are recognized and accepted within current biomedical research communities. Recognizable criteria and clear evidence are required as scientific proof, but how can these be reconciled with the markers and modes of evidence traditionally used in Chinese medicine? Researchers endeavor to honor the characteristics of Chinese medicine while simultaneously seeking the affirmation of the international scientific community. Our studies of classical medical literature in China reveal that in pre-modern times, practitioners of Chinese medicine primarily used two methods for evaluating effectiveness [1]. The first was oral reports of subjective observations of patients regarding their experiences and feelings; the other was physician’s observations and evaluations to determine if these signs and symptoms indicated pathological changes. Physicians were particularly focused on determining whether the overall condition was moving towards resolution or exacerbation. The subjective sensations and observations of both the physicians and the patients were given primary significance in this system. However, for obvious reasons, neither of these sets of “evidence” has been objectively quantified and therefore it is still difficult to incorporate them into modern, scientific research. Our research team is focused on both, and one of the keys is to explore in-depth the subjective feelings. An analysis of practitioners’ perceptions is indispensable for insight into how to evaluate the effect of the subsequent patient visits and make the following treatment decisions. In this article, we used face-to-face interviews to gather data about TCM practitioners’ perceptions in order to refine and develop domains which are the foundations of measurement.

## Background

According to the World Health Organization International Clinical Trials Registry Platform (WHO ICTRP): “Outcomes are events, variables, or experiences that are measured because it is believed that they may be influenced by the intervention. [2]” Research shows that evaluating the effectiveness and safety of clinical interventions depends upon many different factors, including but not limited to patient-reported outcomes (PRO), clinician-reported outcomes (CRO), laboratory tests and medical device measurements [3].

The United States Food and Drug Administration (USFDA) define PRO as follows: “A PRO is any report of the status of a patient’s health condition that comes directly from the patient, without interpretation of the patient’s response by a clinician or anyone else. [4]” During the initial intake, patients provide the most essential information regarding their prior behavior, their state of health and their general quality of life.

During follow-up appointments, PRO helps evaluate the effectiveness, disease progression and disease prognosis [5]. The concept of PRO has caught the attention of the Chinese medicine community because it seems to fit the traditional emphasis on subjective patient reporting that we find in Chinese medicine. Since ancient times, practitioners of Chinese medicine have used the patient’s chief complaint as the main indicator of treatment outcomes. For this reason, PRO has been adopted by researchers of Chinese medicine who see it as a viable method for creating an evidence base for traditional Chinese medicine. Researchers in China have also experimented with other ways of modifying methodology to better suit the characteristics of Chinese medicine [6–10].

CRO is also a technical term proposed by researchers in Western medicine. Our review of the literature shows that in clinical practice, Western medical doctors primarily use three forms of CRO. The first is reporting the signs and symptoms of the patient after treatment, the second is the physician’s evaluation and interpretation of lab reports, and the third is utilizing survey instruments specific to the condition and its presentation [11]. Each of these may reflect the curative effects of the interventions.

It is no exaggeration to say that Chinese medicine relies almost exclusively on PRO and CRO for the evaluation of treatment outcomes. PRO in TCM is based on the patient’s subjective sensations and experiences, while CRO is primarily a description of practitioners’ perceptions throughout treatment. Although these two may overlap, they are always distinct in some aspects [12].

PRO is almost identical in Chinese and Western medicine. CRO, on the contrary, is rooted in the principle of “determining treatment in accordance with patterns identified” and is what makes Chinese medicine a distinct, recognizable traditional medicine. In TCM this process is often called “treatment based on pattern differentiation”. Ultimately, pattern differentiation is essential for achieving therapeutic effects. Identifying patterns based on the variety of signs and symptoms that any one patient may be presented with requires clinical skill and expertise. We can define a ‘pattern’ as an abstract description of the pathological state, and its related signs and symptoms, from the holistic perspective TCM theory [13]. ‘TCM pathogenesis’ means that the doctor grasps the relationship between external manifestation and internal essence, and can relate them directly to the occurrence and development of diseases [14]. In order to pick an appropriate treatment, the clinician needs to collect the signs and symptoms to interpret how the human body may be in disharmony and determine the underlying patterns. On subsequent visits, the physician can perceive an individual’s particular pattern of disharmony, gauge improvement based on severity of symptom, and further assess additional patterns and tendencies that exist at that point in time. He or she will then modify the prescription based on the patient’s status at that moment.

Although CRO have been highly-valued in TCM for thousands of years, they have not yet been the subject of detailed systematic research. Similar to PRO, the initial stage of research involves the articulation of a conceptual framework that fits the unique characteristics of CRO in Chinese medicine. Effectiveness and safety are the main content of CRO. In addition, we found that ‘satisfaction’ is an important aspect in Chinese medicine [11], which sets it apart from Western medicine. ‘Satisfaction’ in CRO in TCM will in turn have an effect on the next clinical decision. TCM physicians usually do not change the prescriptions when there have been satisfactory therapeutic effects. However, if the effects are not significant enough, the practitioner will either modify the original formula if he or she believes the pattern discernment was correct, or change the patterns and the prescription accordingly. Based on our preliminary results, we suggest that “Chinese medicine CRO is closely based on the description of pathogenesis, and captures its general trend towards exacerbation or amelioration, along with other signs and symptoms. This is the basis for prognosis and clinical decisions about further interventions”. Using this as a working hypothesis, we designed semi-structured interviews and interviewed TCM practitioners in person to explore how they understand the results of their process of treatment based on pattern differentiation. Exploration of the potential relationships of effectiveness, safety and satisfaction was designed to further refine the definition of Chinese medicine CRO and its application.

## Materials and methods

Semi-structured interviews were conducted at three university-affiliated hospitals and two scientific institutions in order to collect qualitative data on practitioners’ perceptions of TCM treatment outcomes. These sites were selected to facilitate recruitment of experienced practitioners. Although “experienced” can be defined in various ways, TCM doctors possessing a senior professional title were selected for this study because they generally have extensive experience in clinical medicine and/or research. A qualitative inquiry approach was used that emphasized personal explorations of TCM treatment effects, evaluation of clinical outcomes and processes for treatment decisions. Demographic data was collected near the end of the interview. Informed consent was obtained from every subject at the beginning of the interview.

### Setting

Interviews were conducted in each doctor’s office and were conducted individually by one researcher. An appointment was made with each interviewee in advance to ensure that the interview was carried out smoothly with no interruption or disturbance. The researcher was responsible for managing the interview process, ensuring that all topics on the agenda to be discussed were covered within the interview time.

### Population and recruitment

Nine senior TCM practitioners were recruited by invitation based on a convenience sampling strategy. The selected samples were chosen to be as representative and diverse as possible in terms of hospital degree, division, educational background and professional experience.

TCM is a form of clinical medicine that relies heavily upon empirical observation. All of the participants are well-respected by their peers in the field of TCM, and all had very busy practices with their respective institutions. Three participants (two specialists in acupuncture and one in spleen-stomach diseases) were from Guang’anmen Hospital of China Academy of Chinese Medical Sciences (CACMS); two others (one specialized in gynecology and the other in lung diseases) were from the First Affiliated Hospital of Heilongjiang University of Chinese Medicine; two other pediatric practitioners were from Dongfang Hospital of Beijing University of Chinese Medicine; another one specialized in acupuncture at the Institute of Acupuncture and Moxibustion at CACMS; and another one had expertise in heart diseases and worked at the Institute of Basic Research in Clinical Medicine of China Academy of Chinese Medical Sciences.

### Data collection

One-on-one interviews were conducted. The interview sessions averaged approximately 27 min and were audio recorded. Participants received a small honorarium to express our gratitude for their participation. Recordings were transcribed, de-identified, and imported into ATLAS.ti, version 6 for qualitative analysis.

To characterize the participants, demographics were collected through a written survey at the end of the interview. Qualitative data was collected by conducting semi-structured face-to-face interviews with TCM practitioners at the five medical institutions. As mentioned, an interview guide was developed to elicit interviewee’s opinions from the perspective of effectiveness, safety and satisfaction. Domains of inquiry included perceptions about knowledge of effectiveness, transformation of pathogenesis, causes of adverse reactions and practitioners’ satisfaction with experiences. The following questions were asked:

*1. How do you evaluate treatment outcomes in clinical practice?*

*2. How do you understand the relationship between the transformation of pathogenesis over time and effects of treatment?*

*3. Are there any particular signs that help you to identify the primary transformation of pathogenesis?*

*4. What do you think new signs or symptoms emerging during treatment mean?*

*5. How do you know whether the results after the treatment as reported by the patient or observed by the practitioner represent efficacy, adverse reactions, or neither?*

*6. Do you usually have a specific prognosis in mind when you make a prescription?*

*7. If the actual effect is different from what you expected, how does this affect your follow-up treatment?*

*8. For follow-up patients what is the relationship between the current prescription and the previous one?*

Guides were used to provide a semi-structured approach and allow flexibility to uncover topics presented by the participants not included in the field guide questions.

### Analysis

Demographic data was input into SPSS version 16.0 for analysis. Characteristics of interviewees were described including gender, age, academic background, professional title, specialty and years of experience.

**Fig. 1:**
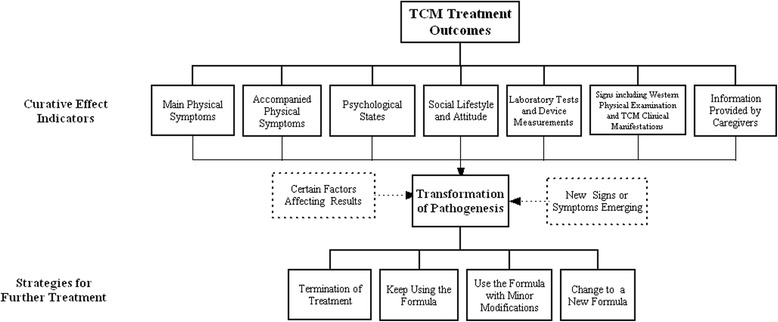
Ways of TCM practitioners report treatment outcomes

Qualitative analysis began with development of a priori code list based on the central research question: "How to understand treatment outcomes based on pattern differentiation?" The conceptual model domains constructed from the results of previous literature research specifically looking at perceptions of TCM providers provided a starting point for identifying the code list. Investigators immersed themselves in the data by reading the transcripts multiple times. Our organizing phase consisted of one researcher applying the a priori code list and augmenting it with free coding. From this, uniform coding sheets were independently coded by two of the investigators and a research assistant. As coding progressed, new codes were added to augment the original list and redundant codes were merged by agreement. Codes were reviewed iteratively by all three coders using inductive content analysis methods to achieve consensus. Inductive content analysis is a method that allows researchers to build models to describe process determining treatment in accordance with patterns identified by TCM practitioners in a conceptual form (Fig. 1). Discrepant codes were discussed on a case-by-case basis. Emerging themes were categorized, abstracted, and discussed. ATLAS.ti mapping features were used to explore super-domains and to aggregate codes into themes. Pertinent and illustrative quotes were then selected for inclusion in our results and further analyzed in our discussion.

## Results

### Participant characteristics

We made the assumption that there is a correspondence between doctors with busy practices (i.e. a large volume of patients compared to other doctors in the same institution) and patient satisfaction. We further assumed that clinical efficacy, at least as perceived by the patient, is the main source of patient satisfaction. It seems reasonable, then, to assert that doctors who are popular in a particular community are effective at clinical pattern differentiation and in making effective prescriptions.

Three of the participating practitioners were male; the average age was 47.6 years (Table 1). One practitioner had been engaged in gynecology for three years. Another two had worked as advanced pediatric practitioners over 20 years. Another three had expertise in acupuncture, two of them for more than 30 years and one for 15 years. The other three practitioners specialized in cardiovascular, respiratory and digestive functions, respectively. There were seven participants who hold PhD degrees, and the other two held master and bachelor degrees respectively. Three participants had concentrated on “integrated Chinese and Western medicine” in their formal studies, and the others studied TCM only.

**Table 1:** Characteristics of interviewees

	Practitioners *N* = 9
Gender	
Female	6 (66.7)
Male	3 (33.3)
Academic background	
TCM	6 (66.7)
Integration	3 (33.3)
Education background	
Bachelor’s	1 (11.1)
Master’s	1 (11.1)
Doctorate	7 (77.8)
Professional title	
Associate chief physician	2 (22.2)
Chief physician	7 (77.8)
Physician specialty	
Internal medicine	3 (33.3)
Acupuncture	3 (33.3)
Gynecology	1 (11.1)
Pediatrics	2 (22.2)
Years of experience	22(3–37)
Interview length (minutes)	27(10)

Results are number (%) for all variables, except median (range) for years of experience and mean (sd) for interview length.

### Qualitative results

The qualitative responses of participants provide context for the treatment outcomes and insight into pattern differentiation. Several themes emerged which, together, characterized the evaluation of sequential treatment outcomes as an important part of the process of individual treatment based on pattern differentiation. Themes included the following: (Theme 1) ways of evaluating treatment outcomes; (Theme 2) relationships between treatment outcomes and pathological transformation; and (Theme 3) distinguishing manifestations of the healing process from true adverse reactions. These themes distilled into the determining treatment principles for further follow-up (Table 2, Theme 4). Finally, an additional theme (Theme 5) emerged related to the characteristics of diagnosis and treatment in TCM.

### Theme 1: Ways of evaluating treatment outcomes

Several different methods were used to evaluate treatment outcomes based on pattern differentiation. These included evaluating primary and co-existing physical symptoms, psychological states, and social lifestyle and attitude based on patients’ feedback. All interviewees were of the opinion that the evaluation of patients’ overall health condition is indispensable in TCM. This means that TCM treatment outcomes were concerned with understanding patients’ entire bio-psycho-social health, not just a single disease or symptom. Typical examples of informants’ comments are as follows:

Relief from the patients’ chief complaints is the main criteria for evaluating effectiveness. The patient often takes the initiative in telling us what he is concerned about and most anxious to improve, which is the most important part of assessment of therapeutic effects*.*

Concomitant symptoms are also very important, such as sleep, diet, urine and stool… Sometimes, it may be advisable to direct your focus to some concomitant discomforts first. For example, treatment to increase appetite, promote bowel movements and improve sleep, will often lead to an improvement in the patient’s overall health. One can then direct attention to resolving the chief complaint. One often gets better results by proceeding in this manner. When evaluating curative effects, one must also consider other symptoms*.*

TCM treatment has beneficial effects on patients’ general well-being and functioning. When physical symptoms are relieved, moods usually also improve, and patients' happiness index increases. This creates a continuous positive feedback*.*

Doctors valued not only the subjective experience, but also the objective signs during a clinical encounter, which provided the basis for understanding the relationship between pattern, principle and prescription. The most common terms used to describe signs included “mind,” “energy,” “spirit,” “soul,” “tongue picture,” and “pulse manifestation.” The following quote also reflects the value placed on signs and its influence on continuity of care:

When the patient came in for follow-up, I consider whether his or her complexion is sallow, pale or normal; whether he is able to express himself clearly, accurately, and loudly, or whether he cannot explain himself well. Based on this information, I can make a preliminary judgment about whether the previous treatment was effective or not. In addition, it is very important to observe the variations in tongue picture and pulse condition to guide the clinical therapy. I think all practitioners in TCM have reached consensus on this point*.*

Several doctors reported that, biomedical examinations were also significant components of treatment outcomes. Two pediatricians and another respiratory physician especially mentioned that, in some cases, it was necessary to “listen to the lungs with a stethoscope” or to “look at the throat using a tongue depressor” in order to judge the effects before and after treatment. Nowadays, lab tests and other technical measurements from Western medicine are widely used in modern clinical practice of TCM. All participants agreed that, TCM treatment could also result in the change of physiological outcomes. Results from laboratory and imaging departments were then used as important references for evaluating curative effects.

Participants also expressed the importance of caregivers, who provided some significant information when patients were unable to respond for themselves accurately. For example: “For some patients with mental disorders, I usually ask their caregivers perceptions of therapeutic effect; for the babies and children, their parents’ reports may be more meaningful. Because sometimes the caregivers’ statements are in disagreement with the patients’ statements, I have to take them all into account synthetically, including the patients, the caregivers, the labs and myself in the process.” Another example:

Caregiver reports are not the only factor in determining effects, and different kinds of diseases require different emphases. The improvement of symptoms is the most important factor in the treatment of acute diseases, but for chronic diseases, the changes in the tongue picture, pulse manifestation, psychological and social functioning are more meaningful for treatment assessment than that of the main symptoms*.*

Another important element described by practitioners was patients’ compliance with treatment. Generally speaking, it was only when the initial result was satisfactory that patients were willing to accept follow-up treatment. Therefore, the presence of patients at a follow-up appointment was in part a sign of the effectiveness of the initial treatment.

However, we cannot entirely rule out the possibility that some of the benefits might come from the so-called placebo effect through contact with the practitioner. More trust and better cooperation generally lead to more satisfaction and adherence. “Those who are more relaxed are more open and forthcoming with their physicians may have better outcomes as a result.” When the actual effect did not meet expected goals, doctors then considered all the possible reasons why. A key informant stated: “Sometimes, when patients have no obvious improvement, it is not because the treatment is not appropriate, but because the treatment could not overcome other factors adversely affecting the patient. These factors include stress, poor sleep and other lifestyle factors. Because the human body and the universe have some similarities from the TCM perspective, no amount of treatment could eventually compensate for the damage incurred from lifestyle.” Overall, practitioners felt TCM treatment outcomes provided them with a valuable reference that helped them make decisions and guide the process of pattern differentiation and individual treatment.

### Theme 2: Relationships between treatment outcomes and pathological transformation

Practitioners commented on the uniqueness of treatment outcomes in TCM. Examples included detailed interpretation of pattern and pattern differentiation or impressions of pathogenesis transformation that hold implications for long-term treatment. “Patterns were like a snapshot in time and reflect the state of the body at a particular stage of the patho-dynamic process.” A participant described the process of TCM treatment in detail:

In order to pick an appropriate treatment, I need to collect the signs and symptoms to interpret how the human body might be in disharmony and determine the relevant patterns. On the subsequent visit, I could visualize an individual’s particular pattern of disharmony, gauge improvement by symptom severity, tell what other subtle tendencies exist and implement other prescriptions based on the patient’s status. I need to understand the ways in which the pathogenesis has shifted from one state to another state and track the state changes to make treatment decisions. In fact, distinguishing changes of pathogenesis is to differentiate patterns*.*

Other examples of treatment outcomes were very specific, i.e., tracing the pathological evolution in order to predict whether the condition is getting better or worse and change the treatment flexibility based on this. Practitioners use their perception continuously to discern patterns and form a corresponding treatment to obtain harmony in the patient. In one participant’s words, “What is so interesting about pathological transformation is the fact that it’s a different viewpoint from the one you originally had and that allows you to understand your prescription and outcomes deeply.”

More specifically, pathological transformation that occurred during the treatment period helped physicians recognize and perceive the associations between body status, prescription medication, and a subsequent, beneficial impact on their discernment of the patterns from a new viewpoint. As one of participants said: “The more carefully you examine your patient, the better you will understand the potential pathological tendency... attempting to modify the treatment principle, make a proper prescription that is good for the patient… It makes a lot of sense during the clinical encounter.”

For some physicians, pathological transformation was emphasized most in doctor-reported concepts, but had never been explored before although they existed objectively. All interviewees expressed an important point that pathological transformation was not at the same level with treatment outcomes. One physician particularly described it as thus:

Through patients’ complaints, laboratory test results, combined with my own observations and examinations, I can make a comprehensive evaluation to determine the process of the disease. Pathological transformation is a synthetic assessment based on treatment outcomes and clinical manifestation. Those signs and symptoms associated with pathological transformation cannot be used to judge whether the therapies were effective or not, because the transformation has the possibility of improving or worsening*.*

Most doctors agreed that the basis of making decisions about treatment adjustment is the dynamic pathological evolution, with later decisions being determined by referring to the previous ones. Only one physician expressed an alternative perspective. He said, “Under certain circumstances, it may be suitable to know what the result is from the past treatment, but in the majority of cases, it is better not to do so because determining pattern differentiation without being influenced by any other factors tends to lead to better effectiveness. Furthermore, if I always use the original prescription or modification, I sometimes may be regarded as not fully responsible. Because the state of the body is always in a process of dynamic change, and therefore different at different points in time, I prefer to not get any outside influence, and take a new look at the patients’ health status myself. This is consistent with the principles of TCM diagnosis and treatment.”

### Theme 3: Differentiation of effectiveness from adverse reactions

Practitioners stated adverse reactions almost never occur in TCM treatment. If the patient’s presentation after treatment wasn’t in accordance with what was expected, one would first consider the genetic factors. For instance, one participant stated:

It’s almost hard to say the causes of treatment. Generally speaking, if the patient feels discomfort after the first dose and grows worse gradually after the following doses, it may be due to the treatment. I think the patient’s discomfort is rarely due to just one or two doses of herbs. There are probably other factors that affect the treatment outcomes, such as catching a cold, feeling anxious, staying up late, getting angry, etc. If the treatment is not effective after a short time, and other factors have been excluded, a weak condition could be a potential reason*.*

Most of the participants we interviewed reported that they paid more attention to the emerging symptoms and emphasized distinguishing between effectiveness and adverse reactions. If the presentation coincided with the typical course of disease, the treatment was likely to be effective in reversing adverse consequences. Most stated that both their clinical experience and medical literature should be used to understand this phenomena. One pediatrician took diarrhea as an example:

Purgatives can lead heat downward… If the child shows slight diarrhea after taking the decoction, I will tell his parents it is normal and is in fact an indication that the treatment is effective*.*

Nevertheless, once it had been confirmed that the result was adverse in some way, the reasons must be analyzed. All of them recognized that they had to first consider whether it was due to inaccurate pattern differentiation, which was the most common cause of ineffectiveness. The solution is to adjust the diagnostic reasoning behind pattern differentiation. Beyond that, all of them believed misuse of herbs, such as inappropriate compatibility, inferior quality and improper decocting procedures, etc., were all possible causes of physical discomforts. Some physicians also believed that Chinese medicine combined with Western medicine can easily cause adverse reactions. In addition, some physicians mentioned that they also had to take into consideration whether there were other drugs being used concomitantly to treat other diseases.

### Theme 4: Optional treatment principles of further follow-up

The principles for further follow-up, summarized in Fig. 1, included terminating treatment, continuing the last formula, using the formula with a little modification and changing the treatment principles to write a new formula.

All physicians said that when patients generally felt well and their condition was stable, treatment should not be discontinued immediately. However, it depended on the circumstances. For acute diseases, this could be regarded as a basic cure and one could terminate treatment. But for chronic conditions, they would still need a few additional sessions to consolidate the results. Two participants discussed the insight they gained after evaluating treatment outcomes and its impact on the following treatment:

It is suitable when there is a fever, such as with the flu, to stop treatment immediately upon recovery. But for coughing, due to chronic conditions such as asthma, the prescription should be taken for a period of time, especially if the condition has not completely resolved. If the results of the therapy are not satisfactory during this time, it is necessary to take some measures to adjust the prescription. These measures cannot be known with certainty and vary in each individual*.*

Participants also explained the principles of how to adjust prescription. All physicians acknowledged that follow-up treatment reflects their satisfaction with the previous treatment outcomes, at least to a certain extent: “When the patient feels no better, or even feels worse, there is no agreement regarding further treatment strategy. I often have some reasons of my own for adjusting treatment. Opinions from every physician may be different.” Another informant shared:

I have to say that, you know, when it happens, there are many problems to be studied and many measures to be taken, but they must be acted upon carefully. Determine whether to continue, modify, or change the prescription, or change the treatment principle and methods based on pattern differentiation depends on one’s perspective. If one is sure that the previous pattern differentiation and treatment are accurate and appropriate, it seems reasonable to continue using the previous prescription, or modify it slightly, or change to a more appropriate one in order to get the desired effects. Otherwise, it is important to adapt thinking and treatment to the new circumstances. In short, this is a matter of opinion*.*

Practitioners also discussed the role of the past treatment in supporting the further treatment and specific ways in which pattern differentiation supported individual treatment strategies. One doctor stated: “I refer to the previous treatment to get more information about the patients’ condition.” However, another one, who was mentioned above, highlighted the importance of self-judgment: “Although the past treatment is important, sometimes it may be of little help to the decision-maker.” Therefore, optional principles may need to be incorporated into the patient’s treatment and it is hard to generalize about this.

### Theme 5: Characteristics in the diagnosis and treatment of TCM

Discussion of desired outcomes also uncovered an unprompted theme—specific characteristics of TCM diagnosis and treatment that are significant, in evaluating treatment outcomes. When participants were asked to discuss their experience in assessing clinical outcomes, a lot of information about diagnosis and treatment emerged, and the information also stimulated the creation of a picture of what they most valued in their doctor-patient interactions. The majority of participants reported treatment was directed primarily at relieving patients’ main complaints, so the improvement of principal symptoms was the most important treatment outcome. Nevertheless, as a point of comparison, some diseases, such as cancer, were frequently treated with the goal of harmonizing the basic physical functions to improve the quality of life, including eating, drinking, bowel movements, sleeping, etc. One participant expressed the importance of combining methods during the treatment process. He said, “I think it is beneficial to combine pattern differentiation with disease differentiation; this is an important method for improving the therapeutic effect. For example, if a patient is coughing persistently, and if I know the causal factors from a biomedical standpoint first, the treatment I choose might be more effective. It is valuable to confirm whether coughing has been caused by respiratory diseases or by a stroke, because this is helpful for further pattern differentiation.”

## Discussion

Research that increases our understanding of how practitioners perceive TCM treatment outcomes and to what degree they use their assessment of pathological changes to support effectiveness evaluation may provide important insights into the clinical decision-making. Additionally, research that helps health care providers understand the role of CRO in TCM is critical to better characterize the differences between TCM and Western medicine in evaluating treatment outcomes. In general, “best practitioners” are more important than “best practices” in Chinese medicine due to the role of individual judgment. In Western medicine, the standardization of best practices fits the basic practice methodology used by physicians.

Because different TCM practitioners tend to look at the same patient from different points of view, it is possible to make different prescriptions for the same person [15]. How then do we assess the effects of treatment? On one hand, the patient’s feedback is more important than any other factor. In this study, practitioners shared examples of the most common ways in which treatment effects are assessed. Although a variety of methods were used, first-hand data from patients was the most reliable and significant information for evaluating the effects of TCM treatment. Consistent with the findings by our group regarding PRO and TCM [6], PRO could be used in evaluation of therapeutic effect. On the other hand, although different TCM doctors understand the condition of the same patient differently, they could all be effective. TCM doctors examined patients by themselves, such as traditional pulse and tongue picture, to complement treatment assessment. It is important to note that doctors’ reports are very individualized, and closely associated with each doctor’s clinical experience and academic background. It is because CRO only reflect particular sessions during a course of treatment, and do not have the same status as PRO. CRO cover multiple aspects like including the previous curative effects, the previous treatment methods and the next therapeutic strategies. It is valuable for exploring TCM doctors’ overall theoretical understanding.

Objective outcomes are very important in western medicine, and are meaningful in TCM as well. Sometimes, TCM treatment could also influence the results of laboratory tests and medical device measurements vary. There are times when the patient is feeling better, but the objective physiological results as assessed through labs and imaging have not changed. The integrative approach which includes PRO, CRO, and other outcomes from various resources is used to judge curative effects and is widely used in the clinical encounter. In many cases, participants described their experience of how to evaluate treatment outcomes in a comprehensive manner. However no studies yet demonstrate the difference that various approaches make in actual treatment outcomes. The comparison of clinical outcomes may help us to (1) understand the expectation of care providers and patients; (2) provide a model of holistic thinking for other care providers; (3) monitor physical and chemical indicators related to symptoms improving; (4) find the best method for achieving optimal treatment outcomes and (5) explore the management methods of outcomes.

In addition, we found that practitioners speculate pathological tendency according to various outcomes sources. This finding is reinforced with our qualitative findings regarding the significance of the practitioners’ understanding of pathological transformation during the process of TCM treatment. Practitioners synthesize information from various sources to determine how the pathological condition has changed and make further treatment decisions based on it. It’s worth mentioning that pathological changes are only correspondence of treatment outcomes, but cannot directly reflect the effectiveness. Our findings demonstrate that the pathological dynamic is the key to treatment decision in Chinese medicine. Most practitioners placed great importance on evaluating and re-evaluating the pathological dynamic as the most significant etiology and guide to selecting treatment. More research needs to be done to confirm or dispel whether this actually results in better treatment outcomes.

**Fig. 2:**
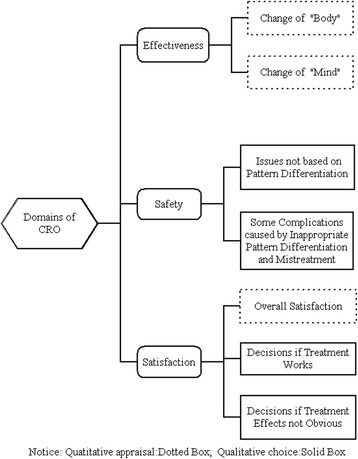
Preliminary domains of CRO in TCM

According to the interviewees’ opinions elicited from the points of effectiveness, safety and satisfaction, it is concluded that CRO measurement could be a combination of qualitative choice and quantitative appraisal. “Effectiveness” is the key part of CRO in TCM, and it was often measured as the overall health status of the patient. It has been suggested that harmony must be observed through both “body” and “mind”. “Safety” is primarily a lack of adverse consequences, including some complications caused by inappropriate pattern differentiation and mistreatment. “Satisfaction” is an overall assessment as well as a marker used in decision-making for the follow-up treatment. The preliminary domains of CRO in TCM have been described in Fig. 2. In future research, we would like to put forward a concrete indexing system and develop a set of treatment effectiveness evaluation questionnaires that distinguish between patient and practitioner evaluations. By comparing outcomes reported by patients and practitioners through the different sessions of treatment, we could lay the foundation for individual evaluation and outcomes management in TCM.

### Strength and limitations

The strength of this study is its use of qualitative methods to provide insight into previously unexplored issues surrounding treatment outcomes from the physicians’ perspective. The use of semi-structured interviews allowed the exploration of several “core” topics with each of the interviewees. Considering the lack of data about the methodological aspects regarding the evaluation of treatment outcomes by TCM physicians, it seems that the results achieved in this study can be considered relevant scientific evidence.

One limitation was that the interviewer had prior knowledge of treatment outcomes, which could have influenced data collection. This was mitigated as much as possible through the use of a topic guide that encouraged the use of non-leading questions. The recruitment methods meant the participants in this study were self-selected. The results could be particular to this (China-based) sample and may not be readily transferred to dissimilar groups and research contexts. The interviewees were, however, recruited from higher-level university-affiliated hospitals and scientific institutions, and were selected to capture a range of professional backgrounds and include physicians with considerable clinical experience.
